# Health Treatment Cost of Holsteins in Eight High-Performance Herds

**DOI:** 10.3390/ani13132061

**Published:** 2023-06-22

**Authors:** Michael R. Donnelly, Amy R. Hazel, Leslie B. Hansen, Bradley J. Heins

**Affiliations:** Department of Animal Science, University of Minnesota, Saint Paul, MN 55108, USA; donne099@umn.edu (M.R.D.); haze0025@umn.edu (A.R.H.); hanse009@umn.edu (L.B.H.)

**Keywords:** health costs, Holstein, mastitis, reproduction

## Abstract

**Simple Summary:**

Health treatments of Holsteins were recorded over a 7-year period and partitioned into five categories (mastitis, reproduction, lameness, metabolic, and miscellaneous). The fixed cost of health treatments was obtained from the veterinary clinics that serviced the eight herds, and they were assigned to each observation. The health treatment cost of cows was highest during the first 30 days in milk for all parities and ranged from USD 22.87 in first parity to USD 38.50 in fifth parity. The total health treatment cost in first parity ranged from USD 23.38 to USD 74.60 for the eight herds and usually increased with parity.

**Abstract:**

Health treatments of Holstein cows (*n* = 2214) were recorded by the owners of eight high-performance dairy herds in Minnesota. Cows calved from March 2008 to October 2015, and 14 types of health treatments were uniformly defined across the herds. Specific types of health treatment were subsequently assigned a cost based on the mean veterinary cost obtained from the veterinary clinics that serviced the eight herds. A fixed labor cost for time (USD 18/h) associated with specific types of health treatment was determined based on interviews with the herd owners and was added to the veterinary cost. Health treatment cost was then partitioned into five health categories: mastitis (including mastitis diagnostic test), reproduction (cystic ovary, retained placenta, and metritis), lameness (hoof treatments), metabolic (milk fever, displaced abomasum, ketosis, and digestive), and miscellaneous (respiratory, injury, and other). Lactations of cows were divided into six intervals that corresponded with stage of lactation based on days in milk. The first interval of lactation was 30 days in length, followed by four intervals of 60 days each, and the final interval started on day 271 and had variable length because it continued to the end of lactation and included the dry period. Health treatment cost was summed within each interval of lactation and subsequently across lactations by parity. Statistical analysis by parity included the fixed effects of herd, interval, and the interaction of herd and interval, with interval regarded as a repeated measure of cows. Health treatment cost was highest during the first interval for all five parities of cows and ranged from USD 22.87 for first parity to USD 38.50 for fifth parity. Reproduction treatment cost was about one-half of the total health treatment cost during the first interval in all five parities. Metabolic treatment cost during the first interval ranged from USD 3.92 (in first parity) to USD 12.34 (in third parity). Compared to the other health categories, mastitis treatment cost was most evenly distributed across intervals of lactation in all parities. Lameness treatment cost was highest during mid- or late-lactation across parities and reflected the time when cows received routine hoof trimming. Additionally, treatment cost across health categories was summed across intervals of lactation for each cow, and the total health cost of cows varied substantially from herd to herd and ranged from USD 23.38 to USD 74.60 for first parity and usually increased with parity.

## 1. Introduction

Historically, selection within the Holstein breed in the US has emphasized increased production alongside conformation traits such as shallower udders, increased body size, and greater dairy form [1,2]. These traits are highly heritable and dairy producers have chosen to optimize production of their cows with the intent of maximizing profit. However, this selection success has been accompanied by a decline in cow health and welfare [3,4,5,6], which can negatively impact the profitability of cows [7]. As a result, selection for improved health is of increasing importance to dairy producers. However, health disorders are difficult to evaluate in the US because uniform recording systems for health data are not utilized, inhibiting the genetic evaluation of health traits [8,9].

Among herds in the United States, three primary software programs are used. The programs are Dairy Comp 305 (Valley Agricultural Software, Tulare, CA, USA), which is used for 35% of herds, followed by PCDART (Dairy Records Management Systems, Raleigh, NC, USA) with 19%, and 15% of herds are examined using DHI Plus (DHI Computing Services Inc., Provo, UT, USA) [10,11].

Many large farms record health data, but health disorders are difficult to analyze because health data lack consistency and are often incomplete [8,9] or are not recorded on large dairy farms [7,12]. For example, some software programs offer tremendous flexibility such that the same health disorder may be entered into the system in different ways. Entries into Dairy Comp 305 tend to be recorded in character acronyms up to four letters, whereas PCDART follows a character acronym format of up to seven letters [13], and Wenz and Giebel [10] reported three to four different acronyms were sometimes used within a single farm to record metritis. Zwald et al. [13] used data from cooperator herds of the Advantage Progeny Test Program from Alta Genetics to assess the health of dairy cows. They found the 724 herds that recorded mastitis did so using 20 various abbreviations via Dairy Comp 305, DHI-Plus, or PCDART. Recently, Hazel et al. [9,14,15] found that utilization of health data for genetic evaluation of common health disorders is possible; however, improving the integrity and uniformity of health data is necessary for genetic evaluation.High-quality health data can contribute to the improvement of management decisions and reduce mortality of cows among herds [16]. Cows with a health disorder early in lactation are at an increased risk of a health disorder later in lactation [17] and knowledge of previous health disorders allows dairy producers to identify cows that may require attention later in lactation. Furthermore, complete and uniform health data can be valuable for minimizing health treatment costs through better identification of health disorders for individual cows as well as for improved culling decisions to remove cows with extensive health disorders from the herd.

The lack of uniformity of recording for health data makes it difficult to summarize health disorders within and between dairy herds. Additionally, when recording health data, dairy producers may not consistently distinguish the difference between incidence and treatment of health disorders. Parker Gaddis et al. [17] reported that incidence rates of health disorders in their study were lower than other literature estimates, which could suggest dairy producers are more likely to record only health treatments rather than to record every health diagnosis.

Genetic evaluations of health traits are conducted in many countries around the world that record routine health data. Commercial dairy companies are offering genetic predictions for health traits of Holsteins [18,19,20] and Jerseys [21]. Several European countries have required recording of all health treatments of dairy cows for over 30 years with health treatments systematically recorded by veterinarians on an individual animal basis [8], which permits the evaluation of health disorders within and between herds. 

Traditionally, selection within the Holstein breed in the US placed major emphasis on production and conformation traits [22]. This approach was accompanied by a decline in cow health and welfare [22], which negatively impacts the profitability of cows. The impact of health disorders on profitability of cows is difficult to measure because most health data recorded on farms lacks uniformity and is often incomplete. Furthermore, most herd owners do not record the cost of individual health treatments of cows. Determining the full economic impact of heath disorders is complicated because health disorders impact involuntary culling, fertility, and production, which in turn influence profitability [13].

The majority of metabolic and infectious health disorders occur within the first 30 to 60 days of lactation [17,23,24], and are caused by the negative energy balance created by the demand for nutrients to produce milk [25]. In a summary of previous studies, Parker Gaddis et al. [17] reported lactational incidence rates were highest for mastitis, lameness, and metritis, and incidence of mastitis ranged from 0.96% to 39.13% with a mean of 17.98%, metritis ranged from 1.77% to 35.50% with a mean of 12.34%, and lameness ranged from 2.54% to 30.44% with a mean of 9.27%. Differences in recording methods, definition of health disorders, and diagnosis protocols have created variability of results from study to study; therefore, incidence rates of health treatments are difficult to compare across studies [23].

Clinical mastitis often results in reduced production [26], and metabolic disorders, lameness, and mastitis tend to negatively impact fertility [27]. Subclinical mastitis, which is more difficult to detect, can reduce production, increase stillbirth, reduce fertility, and decrease profitability of cows [28,29,30,31]. Cobirka et al. [32] reported that many studies reported the cost of a mastitis case averaged over USD 150. Ettema and Santos [33] estimated the cost per case of mastitis to be USD 51, and this included the cost of antibiotic treatment, labor, and 5 d of discarded milk. Guard [34] estimated a much higher cost of USD 224 per case of mastitis when lost revenue from reduced milk production, delayed conception, death, and involuntary culling was included. Jones et al. [35] analyzed the health treatment costs of an experimental herd of Holsteins selected for milk production versus an unselected genetic control line, and the cows selected for milk production had USD 28.22 more health treatment cost in first parity than the control line. In that study, 43% (USD 27.79) of the cost in first parity was attributed to mastitis [35].

Reducing the incidence of health disorders of dairy cows is of growing interest to herd owners. However, SCS (as an indicator of mastitis) and productive life (a composite trait including production and functional traits of cows), are two traits that have permitted selection for improved health of cows in the US [22,36]. Improving the integrity and uniformity of health data recorded on farms will provide an opportunity to assess the economic impact of health disorders and allow for selection of reduced health treatment cost [13,36].

Health disorders impact farm profitability by increasing costs due to veterinary services and pharmaceuticals and decreased milk production [6,9,37,38]. Additionally, cows with mastitis, lameness, or metabolic disorders early in lactation subsequently often have impaired fertility and may acquire additional costs from delayed conception. Some treatments for health disorders have tremendous cost [9,38]; however, dairy producers may have difficulty assigning a cost to specific health disorders, because records may not always encompass the time, the type, and the dosages of pharmaceuticals used for individual health treatments. Health data from commercial dairy herds are often inconsistently recorded and lack completeness; therefore, the determination of cost for health disorders and the inclusion of health traits in genetic evaluations are inhibited.

The assessment of the economic impact of health treatments is rarely undertaken because of the unavailability of appropriate comprehensive and reliable health data of cows. Therefore, the objective of this study was to analyze the health treatment cost of Holstein cows in parity 1 to 5 using on-farm data for treatments that were defined uniformly within 8 high-performance Minnesota dairy herds.

## 2. Materials and Methods

### 2.1. Description of Herds and Cows

Holstein cows in 8 dairy herds throughout Minnesota were enrolled from March to September of 2008 to initiate a long-term study. All the animal procedures involving animal care and management were approved by the University of Minnesota Institutional Animal Care and Use Committee. The herds had the same management criteria for all Holstein cows that included the same criteria for health treatments [14].

Data were lactational health records from Holstein females in parity 1 to 5 for lactations that were initiated from March 2008 to October 2015. In total, 2214 cows with 4979 lactations of variable length in parity 1 to 5 remained for analysis. The distribution of lactation of cows by herd and parity is in Table 1.

### 2.2. Data for Health Treatment and Cost

Health treatments were uniformly defined for 14 individual health disorders across the 8 herds and recorded on-farm with Dairy Comp 305 (Valley Ag Software, Tulare, CA, USA). Researchers for this study had direct contact with the dairy producers to ensure correct assignment of the health treatments and remarks associated with health treatments for completeness of recording. The health treatments were partitioned into 5 categories: mastitis (MAST), reproduction (REPRO), lameness (LAME), metabolic (META), and miscellaneous (MISC). The specific health treatments that were assigned to each of the 5 categories are reviewed in Table 2.

Health treatment costs were based on interviews with the 8 dairy herds and their veterinarians. The veterinary service cost for each of the types of health treatments was obtained from the veterinarians providing service to the 8 herds. Pharmaceutical costs were either obtained from veterinarians or an average catalog cost of pharmaceuticals from 5 veterinary service vendors that serve Minnesota. Therefore, veterinary costs included veterinarian labor, supplies, and pharmaceuticals used for health treatment. The total cost for each specific type of health treatment (Table 3) was determined by summing the respective veterinary cost and the labor cost that was associated with each treatment. Labor cost was assigned a fixed value of USD 18/h, and the time assigned to each specific type of treatment was based on an interview with the 8 herd owners. The labor cost was obtained from interviews with the dairy producers or their employees.

The time within lactation when health treatment cost occurred was of special interest in this study; therefore, lactations of cows were divided into 6 intervals that corresponded to stage of lactation and were based on DIM. The first interval began at calving and was 30 d in length. The subsequent 4 intervals were each 60 d in length (31 d to 90 d, 91 d to 150 d, 151 d to 210 d, and 211 d to 270 d), and the final interval started at 271 DIM and had a variable length because it continued to the end of lactation and included the dry period. Treatments that were routinely administered during the dry period (i.e., vaccinations, dry cow treatments) were not included in the health treatment costs. Very few health treatments occurred during the dry period in these herds, and therefore, the dry period was combined with the last 30 days of lactation. The health treatment cost within each health category was summed to obtain an interval cost by health category. Additionally, the health treatment cost across the 5 categories for a lactation of a cow was, in turn, summed within the interval to arrive at the total health treatment cost (TOT) for that interval. Finally, the TOT for the 6 intervals of lactation were summed to obtain the total lactational health cost (THC) of each lactation of a cow.

### 2.3. Statistical Analysis of Health Cost

Analysis was conducted separately by parity. For analysis of health treatment cost by interval within lactation, dependent variables were the cost of each of health category as well as TOT. Independent variables were the fixed effects of herd, interval, and the interaction of herd and interval, with interval regarded as a repeated measure for cows. An attempt was made during preliminary analysis to fit the fixed effects of year and season of calving; however, they did not significantly account for variation. A separate analysis assessed the THC of cows (dependent variable), and the independent variables were herd as a fixed effect and cow as a random variable. The MIXED procedure of SAS (SAS Institute, 2014) was used to conduct the ANOVA and to obtain least squares solutions. A multi-parity model was considered but may have resulted in biased solutions, because cows with high health treatment cost typically leave herds more quickly than cows with low health treatment cost.

## 3. Results and Discussion

### 3.1. Significance of Effects from Analysis of Intervals

For the analysis of health treatment cost by interval within lactation, the fixed effects of herd, interval, and the interaction of herd and interval were all highly significant (*p* < 0.01) in first parity for each category of health treatment cost as well as TOT. In second parity, the fixed effects of herd, interval, and the interaction of herd and interval were again significant (*p* < 0.05) for each category of health treatment cost and TOT, except not significant (*p* = 0.59) for META cost. In parities 3 to 5, the fixed effect of herd was significant (*p* < 0.05) for each category of health treatment cost, except herd was not significant (*p* = 0.11) for REPRO cost in parity 5. Interval was significant (*p* < 0.05) for each category of health treatment cost and TOT in third parity, but only for REPRO cost, LAME cost, and TOT in fourth parity and only for REPRO cost and TOT in fifth parity. The interaction of herd and interval in third parity was highly significant (*p* < 0.01) for REPRO cost, LAME cost, MISC cost, and TOT but was not significant for MAST and META costs. In fourth and fifth parity the interaction of herd and interval was significant (*p* < 0.05) only for REPRO cost.

### 3.2. Health Treatment Cost for Intervals in First Parity

Least squares means of health treatment cost by category for the 6 lactation intervals in first parity are in Table 4, which also provides the percentage of each category’s contribution to TOT. The TOT was significantly higher (*p* < 0.05) during the first interval at USD 22.87 than all other intervals, and cows accrued the most cost for REPRO, META, and MISC during this interval. The high cost for REPRO and META during the first interval was expected, because these categories are primarily composed of treatments for metritis, retained placenta, displaced abomasum, and ketosis, and these health disorders most commonly occur near calving [13,39].

The REPRO cost during the first interval was mostly metritis treatments that, when analyzed separately from other REPRO costs, accrued a mean cost of USD 9.06. Health treatment cost for META during the first interval was mostly because of displaced abomasum with a mean cost of USD 3.02. In general, REPRO and META costs during the first interval of first parity may have been the result of health disorders from the negative energy balance that often occurs postpartum [25]. Beyond the first interval, REPRO cost mainly resulted from treatment for cystic ovaries, mostly during the third interval, when this health disorder was uncovered via palpation or ultrasound. The META cost for later intervals was mostly for diarrhea treatment.

The high MISC cost during the first interval in first parity is because almost one-half of the MISC cost was attributed to treatment for elevated temperatures without a specific health disorder being diagnosed. The treatment of elevated temperature was mostly due to the herd health practices of one herd owner. About one-third of MISC cost during later intervals in first parity was for elevated temperatures. Other MISC cost during first parity after the first interval was evenly split between respiratory and injury treatments.

The MAST cost was highest during interval 1 and interval 6 in first parity but was evenly distributed across intervals 2 to 5. The higher MAST cost during the first interval is in agreement with Appuhamy et al. [24], who reported a higher incidence of mastitis during the first month of lactation than any other time. A possible reason for the elevated MAST cost during the first interval may have been the decreased immune response to infection the cows often experience during the transition period [40]. Additionally, Green et al. [41] indicated poor hygiene causes mastitis during early lactation and, perhaps, the high MAST cost during the first interval in this study was due to exposure to mastitis-causing pathogens in the heifer rearing or calving facilities. Furthermore, the SCC of cows usually increases later in lactation [42], and this may explain the high MAST cost near the end of lactation during interval 6. From a management perspective, the high MAST cost during interval 6 also may have resulted from the culturing of cows near dry-off to identify cows eligible for selective dry cow therapy [43], which was used infrequently in these herds. Routine dry cow treatments were not included in the health treatment costs of cows.

The LAME cost was significantly higher (*p* < 0.05) during intervals 3 and 6 in first parity and reflected the timing of routine hoof trimming and the resulting treatment for hoof health disorders. The LAME cost during intervals 3 and 6 in this study is in disagreement with Koeck et al. [39], who reported lameness incidences are evenly distributed throughout lactation with slightly higher incidence during early lactation. 

### 3.3. Health Treatment Cost for Intervals during Later Parities

Table 5, Table 6, Table 7 and Table 8 provide the least squares means of health treatment cost by category during the lactation intervals in parities 2, 3, 4, and 5, as well as the percentage of each category’s contribution to TOT. In each case, TOT was significantly higher during the first interval and ranged from USD 24.69 in second parity to USD 38.50 in fifth parity. Distribution of treatment cost for each health category except MAST was similar across intervals in parity 2 to 5. Additionally, standard errors of treatment cost for each health category as well as TOT increased with parity because of fewer cows contributing data in later parities.

The REPRO cost was significantly higher (*p* < 0.05) during the first interval than other intervals in parity 2 to 5. The high REPRO cost during first interval was expected because cows experience transition disorders during this period of time [40]. The especially high REPRO cost during the first interval of fifth parity was mainly from metritis treatments, which when evaluated separately, had a mean cost of USD 16.87. Most of the REPRO cost after the first interval was from treatment for cystic ovaries, and in some instances, from additional treatment for metritis. Metritis can require multiple treatments and may occur at various times during lactation from injury to the reproductive tract or from nutritional deficiencies [44].

The META cost was significantly higher (*p* < 0.05) during the first interval for parities 2, 3, and 4, as it was in first parity, and may have been due to negative energy balance after calving [45]. However, META cost in fifth parity was evenly distributed across intervals. In third parity, specifically, the high META cost of USD 12.34 during the first interval was for displaced abomasum, which accrued a mean cost of USD 8.91 (72% of META cost) during the first interval. Surgery for displaced abomasum during a previous lactation perhaps explains the numerically lower displaced abomasum and META cost in fourth and fifth parities. The META cost after first interval in later parities was overwhelmingly due to treatment for digestive disorders.

The distribution of MAST cost across the intervals in later parities was different from first parity. In later parities, the proportion of MAST cost during intervals 2 to 5 was higher than it was in first parity, because in first parity, MAST cost was numerically highest during interval 1 and during interval 6. The difference in distribution of MAST cost across intervals in later parities perhaps resulted from preventative treatment for mastitis at dry-off during the previous lactation and better management during the pre-fresh period for cows than for springing heifers.

Similar to first parity results, the LAME cost in parities 2 to 5 was typically greatest during interval 3 and interval 6. The LAME cost during intervals 3 and 6 reflected the timing of routine hoof trimming that often resulted in hoof treatment, with treatment for hoof ulcers accounting for the majority of the LAME cost. Other than during interval 3 and interval 6, the LAME cost was usually from treatment for hairy wart.

### 3.4. Total Health Cost by Herd and Parity

The results from the analysis of THC by herd and parity are in Table 9, and standard errors for estimates of THC increased with parity because the number of cows declined with parity. The weighted least squares means of THC of cows based on the number of cows in each herd were USD 54.73, USD 75.56, USD 94.43, USD 100.97, and USD 122.29 from first to fifth parity, respectively. No previous research has analyzed health treatment costs from commercial dairy herds; however, Becker et al. [4] analyzed THC of cows in an institutional herd of Holsteins and reported a mean THC in first parity of USD 41.41 and USD 62.41 for cows selected for small and large body size, respectively. The estimates of THC from that study are comparable to THC in first parity of herds in this study that ranged from USD 23.38 to USD 74.60.

The mean THC was variable across herds in this study and tended to numerically increase from first to third parity for all herds except herd B, which had numerically lower REPRO cost in second and third parity than in first parity. The difference in THC between herds is probably a reflection of alternative management practices to treat health disorders. Herd owners who more closely monitor fresh cows for transition disorders are more likely to detect health disorders, and as a result, may provide treatment. Herd C had the highest THC in parities other than first parity, and herd C had health treatment protocols that were more aggressive than the other herds. Additionally, herd C had the highest milk production of the eight herds, and research has documented that higher milk production is associated with increased health disorders [35]. Herd H tended to have low THC in all five parities, and we suspect this was a reflection of its excellent attention to nutritional requirements and detailed transition cow protocols.

The management conditions and preventative measures and management were different for all herds. Decisions for treatment procedures were similar for herds based on antibiotic and pharmaceutical recommendations. Within herd, the decisions on treatment of animals for health treatments were the same for all cows. Therefore, management conditions, welfare, hygienic conditions, milking procedures, and preventative measures would have been implemented for all cows within a herd. Nonetheless, differences in herd management and use of antibiotic treatments and veterinarian use on farms may have played a significant role in the differences observed across farms. Some farms had low THC per cow across all parities; however, other herds had high THC costs.

The difference in THC for herds may not be a true reflection of the health costs of Holstein cows within herds. Some of the herds monitored cows more thoroughly, with more labor and possibly detected more health disorders of cows. Furthermore, some herds may have treated more minor health issues more aggressively [9]. Some of the herds had activity and rumination monitoring systems [46] which may have assisted the workers on farm to detect more health disorders and more estrus events to increase fertility of cows [47,48,49]. Additionally, herds may have used more preventative treatments, vaccinations, or feed supplements to reduce health disorders of cows [9]. Mean days open ranged from 110 to 136 days for first-lactation cows. Additionally, age at first calving had a mean of 23.9 months and ranged 22.7 to 27.8 months for first-lactation cows. These differences in herds may have affected the results for health treatment costs observed in the current study. This may reflect the divergent management strategies and environments of the eight herds. Considerable variation existed for THC of the eight herds, but health treatment cost was substantial for most of the herds. There may be a relationship between the costs of treating cows that may have been culled versus treating the cows that remained in the herd. Quite possibly the health treatment costs would be greater for cows that were culled versus cows that remained in the herd. Culling of cows and the unprofitability of treatment, low milk production, problems with reproduction, and general unprofitability were not accounted for in the current study. An enterprise analysis of multiple years and data collection from birth to culling or death would determine the health treatment costs of cows that were culled, which was not determined in this study.

This study was the first to document the cost of health treatments using field data from large commercial dairies. Because the data were of high quality, health treatment costs were determined for different stage of lactations in which the five categories of health treatment and TOT incurred. Minimizing REPRO and MAST costs should be the upmost priority of dairy producers to enhance profitability.

Improvement of cow welfare and well-being in dairy herds will reduce the THC of cows in all herds and will provide economic benefit to dairy producers. Optimizing cow welfare is especially important because it demonstrates the dedication of dairy producers to provide consumers with a quality product Applying veterinary cost and labor cost to individual health treatments revealed the economic impact of health disorders on the herds. The approach used in this study to supplement incidence of health disorders with their cost provided the opportunity to better utilize health data for day-to-day management decisions. The integrity and uniformity of health data in this study provided a means for creating variation between daughters of sires for health disorders and, perhaps, would expose genetic control of health traits.

## 4. Conclusions

Comparative analysis of health treatments and health costs of dairy herds may be difficult due to various herd management conditions, and genetics, and recording and methodologies for data. However, the presented research shows the problem comprehensively, using large data sets from practice and documents the costs of treatment.

The highest TOT was during the first 30 DIM (interval 1) in all five parities and was mainly due to REPRO and META costs. The MAST cost was highest during interval 1 and interval 6 in first parity but was more evenly distributed across intervals in later parities. The REPRO cost had the largest economic impact in first-parity cows, but in later parities MAST cost was generally the highest health treatment cost for cows in the eight herds. Weighted herd means of THC ranged from USD 54.73 in first parity to USD 122.29 in fifth parity, but across herds, THC varied substantially for parities.

Costs of health disorders in Holstein cows are very costly for numerous categories of disease. The costs of health treatments are considerably influenced by herd management, labor, technology, and dairy market conditions. Globally, dairy producers are aware that profitability of dairy farming is immensely influenced by the total cost of production, which includes uniform recording of health treatments.

## Figures and Tables

**Table 1:** Distribution of cows by herd and parity.

	Parity				
Herd	1	2	3	4	5
A	394	246	125	51	17
B	227	157	100	44	9
C	427	299	181	88	43
D	402	279	150	74	29
E	179	117	59	15	6
F	152	105	48	17	5
G	250	159	66	31	8
H	183	125	71	30	11
Total	2214	1487	800	350	128
Percentage by parity (%)	44	30	16	7	3

**Table 2:** Specific health treatments included in the health categories.

Category	Abbreviation	Treatment
Mastitis	MAST	Mastitis
		Mastitis diagnostic test ^1^
Lameness	LAME	Hoof treatment ^2^
Reproduction	REPRO	Cystic ovaries
		Retained placenta
		Metritis
		Miscellaneous reproduction ^3^
Metabolic	META	Milk fever
		Displaced abomasum
		Ketosis
		Digestive ^4^
Miscellaneous	MISC	Respiratory
		Injury
		Other treatments

^1^ Mastitis diagnostic test included milk culture and California Mastitis Test (Immucell, Portland, ME). ^2^ Hoof treatment included dermatitis, infectious pododermatitis, foot ulcer, and other hoof treatments. ^3^ Miscellaneous reproduction included abortion treatments, caesarean section, pyometria, uterine disorders (adhesion, mass, prolapse, and torsion), and mummified calf. ^4^ Digestive included clostridium, traumatic reticuloperitonitis, hemorrhagic bowel syndrome, peritonitis, twisted cecum, lack of appetite, or any other digestive treatment.

**Table 3:** Total cost assigned to individual health treatments.

Type of Treatment	Veterinary Cost ^1^	Labor Cost ^2^	Total
	---------------------- ($) ----------------------		
Mastitis diagnostic test	8	3	11
Cystic ovaries	14	2	16
Digestive	34	10	44
Displaced abomasum	256	19	275
Hoof treatment	21	9	30
Injury	3	23	26
Ketosis	24	9	33
Mastitis	22	6	28
Metritis	112	5	117
Milk fever	21	17	38
Miscellaneous reproduction	170	19	189
Other	25	6	30
Respiratory	67	10	77
Retained placenta	75	5	80

^1^ Veterinary cost was obtained from the veterinary clinics that serviced the 8 herds. ^2^ Fixed labor cost (USD 18/h) across the 8 herds.

**Table 4:** Least squares means, standard errors, and percentage of total cost (TOT) by category ^1^ of health cost during 6 intervals of lactation in first parity.

	Health Category																	
Interval	MAST			REPRO			LAME			META			MISC			TOT		
	$\overset{-}{X}$	SE	%	$\overset{-}{X}$	SE	%	$\overset{-}{X}$	SE	%	$\overset{-}{X}$	SE	%	$\overset{-}{X}$	SE	%	$\overset{-}{X}$	SE	%
	----- ($) -----			----- ($) -----			----- ($) -----			----- ($) -----			----- ($) -----			----- ($) -----		
1 (0 to 30 d)	2.78 ^a^	0.21	12	11.78 ^a^	0.47	52	0.84 ^d^	0.19	4	3.92 ^a^	0.38	17	3.65 ^a^	0.23	16	22.87 ^a^	0.74	100
2 (31 to 90 d)	1.55 ^c^	0.21	28	0.65 ^b^	0.48	11	1.39 ^c^	0.19	25	0.89 ^b^	0.39	16	1.15 ^b^	0.23	20	5.63 ^cd^	0.76	100
3 (91 to 150 d)	1.97 ^bc^	0.21	26	1.36 ^b^	0.49	18	2.93 ^b^	0.20	39	0.39 ^b^	0.40	5	0.84 ^bc^	0.24	11	7.48 ^c^	0.78	100
4 (151 to 210 d)	1.71 ^bc^	0.22	40	0.35 ^b^	0.50	8	1.46 ^c^	0.20	34	0.47 ^b^	0.40	11	0.30 ^c^	0.24	7	4.30 ^d^	0.79	100
5 (211 to 270 d)	1.49 ^c^	0.22	27	0.28 ^b^	0.50	5	1.72 ^c^	0.20	31	0.58 ^b^	0.41	10	1.48 ^b^	0.25	27	5.55 ^cd^	0.80	100
6 (271 d to end)	2.22 ^ab^	0.22	22	0.22 ^b^	0.50	2	5.84 ^a^	0.20	57	0.79 ^b^	0.41	8	1.15 ^b^	0.25	11	10.23 ^b^	0.80	100

^1^ MAST = mastitis, REPRO = reproduction, LAME = lameness, META = metabolic, and MISC = miscellaneous. ^a–d^ Superscripts denote significant differences (*p* < 0.05) within each health category (column).

**Table 5:** Least squares means, standard errors, and percentage of total cost (TOT) by category ^1^ of health cost during 6 intervals of lactation in second parity.

	Health Category																	
Interval	MAST			REPRO			LAME			META			MISC			TOT		
	$\overset{-}{X}$	SE	%	$\overset{-}{X}$	SE	%	$\overset{-}{X}$	SE	%	$\overset{-}{X}$	SE	%	$\overset{-}{X}$	SE	%	$\overset{-}{X}$	SE	%
	----- ($) -----			----- ($) -----			----- ($) -----			----- ($) -----			----- ($) -----			----- ($) -----		
1 (0 to 30 d)	1.80 ^c^	0.33	7	12.81 ^a^	0.63	52	1.18 ^d^	0.27	5	5.85 ^a^	0.52	24	3.04 ^a^	0.29	12	24.69 ^a^	1.03	100
2 (31 to 90 d)	3.09 ^b^	0.34	35	0.52 ^c^	0.64	6	2.02 ^c^	0.27	22	1.94 ^b^	0.54	22	1.39 ^b^	0.30	16	8.96 ^c^	1.06	100
3 (91 to 150 d)	4.41 ^a^	0.35	32	1.98 ^b^	0.66	14	4.30 ^b^	0.28	32	1.72 ^b^	0.55	13	1.24 ^b^	0.31	9	13.65 ^b^	1.09	100
4 (151 to 210 d)	4.21 ^b^	0.36	41	1.17 ^c^	0.68	11	2.52 ^c^	0.29	25	1.38 ^b^	0.56	14	0.91 ^b^	0.31	9	10.17 ^c^	0.11	100
5 (211 to 270 d)	3.43 ^b^	0.37	44	0.16 ^c^	0.69	2	2.24 ^c^	0.29	29	0.85 ^b^	0.57	11	1.04 ^b^	0.32	13	7.72 ^c^	1.13	100
6 (271 d to end)	3.03 ^ab^	0.37	28	0.03 ^bc^	0.69	0	6.11 ^a^	0.29	56	0.64 ^b^	0.58	6	1.06 ^b^	0.32	10	10.86 ^bc^	1.14	100

^1^ MAST = mastitis, REPRO = reproduction, LAME = lameness, META = metabolic, and MISC = miscellaneous. ^a–d^ Superscripts denote significant differences (*p* < 0.05) within each health category (column).

**Table 6:** Least squares means, standard errors, and percentage of total cost (TOT) by category ^1^ of health cost during 6 intervals of lactation in third parity.

	Health Category																	
Interval	MAST			REPRO			LAME			META			MISC			TOT		
	$\overset{-}{X}$	SE	%	$\overset{-}{X}$	SE	%	$\overset{-}{X}$	SE	%	$\overset{-}{X}$	SE	%	$\overset{-}{X}$	SE	%	$\overset{-}{X}$	SE	%
	----- ($) -----			----- ($) -----			----- ($) -----			----- ($) -----			---- ($) ----			----- ($) -----		
1 (0 to 30 d)	2.65 ^b^	0.53	8	13.36 ^a^	0.91	41	1.56 ^c^	0.41	5	12.34 ^a^	1.09	38	2.88 ^a^	0.46	9	32.79 ^a^	1.72	100
2 (31 to 90 d)	5.49 ^a^	0.56	49	0.41 ^b^	0.96	4	2.36 ^bc^	0.43	21	1.83 ^b^	1.15	16	1.05 ^b^	0.48	9	11.14 ^bc^	1.82	100
3 (91 to 150 d)	5.27 ^a^	0.60	32	1.53 ^b^	1.02	9	5.18 ^a^	0.46	32	2.66 ^b^	1.22	16	1.65 ^ab^	0.51	10	16.28 ^b^	1.93	100
4 (151 to 210 d)	4.81 ^a^	0.61	36	0.70 ^b^	1.04	5	2.86 ^b^	0.47	22	2.61 ^b^	1.25	20	2.32 ^ab^	0.52	17	13.31 ^bc^	1.97	100
5 (211 to 270 d)	4.09 ^ab^	0.63	46	0.29 ^b^	1.07	3	1.96 ^bc^	0.48	22	1.68 ^b^	1.28	19	0.89 ^b^	0.54	10	8.92 ^c^	2.03	100
6 (271 d to end)	2.84 ^b^	0.63	22	0.47 ^b^	1.07	4	6.38 ^a^	0.48	49	1.90 ^b^	1.29	15	1.30 ^b^	0.54	10	12.89 ^bc^	2.04	100

^1^ MAST = mastitis, REPRO = reproduction, LAME = lameness, META = metabolic, and MISC = miscellaneous. ^a–c^ Superscripts denote significant differences (*p* < 0.05) within each health category (column).

**Table 7:** Least squares means, standard errors, and percentage of total cost (TOT) by category ^1^ of health cost during 6 intervals of lactation in fourth parity.

	Health Category																	
Interval	MAST			REPRO			LAME			META			MISC			TOT		
	$\overset{-}{X}$	SE	%	$\overset{-}{X}$	SE	%	$\overset{-}{X}$	SE	%	$\overset{-}{X}$	SE	%	$\overset{-}{X}$	SE	%	$\overset{-}{X}$	SE	%
	---- ($) ----			---- ($) ----			---- ($) ----			---- ($) ----			---- ($) ----			---- ($) ----		
1 (0 to 30 d)	3.59 ^b^	1.06	12	11.25 ^a^	1.56	38	3.00 ^b^	0.77	10	8.26 ^a^	1.70	28	3.14 ^a^	0.67	11	29.25 ^a^	2.94	100
2 (31 to 90 d)	5.22 ^ab^	1.14	33	2.41 ^b^	1.67	15	3.74 ^b^	0.82	24	3.04 ^b^	1.82	19	1.19 ^b^	0.72	8	15.60 ^b^	3.16	100
3 (91 to 150 d)	7.05 ^a^	1.25	32	3.41 ^b^	1.84	16	7.38 ^a^	0.90	34	2.74 ^b^	2.00	13	1.21 ^ab^	0.79	6	21.79 ^ab^	3.46	100
4 (151 to 210 d)	6.07 ^ab^	1.30	48	0.00 ^b^	1.91	0	3.38 ^b^	0.94	27	2.23 ^b^	2.08	17	1.08 ^ab^	0.82	8	12.76 ^b^	3.60	100
5 (211 to 270 d)	4.93 ^ab^	1.34	40	0.09 ^b^	1.96	1	3.55 ^b^	0.97	29	2.43 ^b^	2.14	20	1.35 ^ab^	0.85	11	12.35 ^b^	3.71	100
6 (271 d to end)	3.54 ^ab^	1.36	28	0.00 ^b^	2.00	0	6.56 ^a^	0.98	52	1.39 ^b^	2.18	11	1.04 ^ab^	0.86	8	12.52 ^b^	3.77	100

^1^ MAST = mastitis, REPRO = reproduction, LAME = lameness, META = metabolic, and MISC = miscellaneous. ^a–b^ Superscripts denote significant differences (*p* < 0.05) within each health category (column).

**Table 8:** Least squares means, standard errors, and percentage of total cost (TOT) by category ^1^ of health cost during 6 intervals of lactation in fifth parity.

	Health Category																	
Interval	MAST			REPRO			LAME			META			MISC			TOT		
	$\overset{-}{X}$	SE	%	$\overset{-}{X}$	SE	%	$\overset{-}{X}$	SE	%	$\overset{-}{X}$	SE	%	$\overset{-}{X}$	SE	%	$\overset{-}{X}$	SE	%
	---- ($) ----			---- ($) ----			---- ($) ----			---- ($) ----			---- ($) ----			---- ($) ----		
1 (0 to 30 d)	5.47 ^a^	1.59	14	20.70 ^a^	2.90	54	1.70 ^b^	1.08	4	8.16 ^a^	2.71	21	2.47 ^a^	1.38	6	38.50 ^a^	4.97	100
2 (31 to 90 d)	4.59 ^a^	1.82	31	2.19 ^b^	3.32	15	2.31 ^b^	1.24	16	3.91 ^a^	3.10	27	1.71 ^a^	1.59	12	14.72 ^b^	5.69	100
3 (91 to 150 d)	6.13 ^a^	1.90	34	4.57 ^b^	3.47	25	3.63 ^ab^	1.30	20	2.10 ^a^	3.23	12	1.80 ^a^	1.65	10	18.23 ^b^	5.94	100
4 (151 to 210 d)	3.82 ^a^	1.92	33	0.00 ^b^	3.57	0	2.67 ^ab^	1.33	23	2.64 ^a^	3.33	23	2.44 ^a^	1.70	21	11.58 ^b^	6.11	100
5 (211 to 270 d)	2.27 ^a^	2.00	39	0.00 ^b^	3.67	0	0.92 ^b^	1.37	16	1.06 ^a^	3.42	18	1.64 ^a^	1.75	28	5.90 ^b^	6.28	100
6 (271 d to end)	4.98 ^a^	2.02	33	0.18 ^b^	3.69	1	6.03 ^b^	1.38	41	3.14 ^a^	3.44	21	0.55 ^a^	1.76	4	14.88 ^b^	6.32	100

^1^ MAST = mastitis, REPRO = reproduction, LAME = lameness, META = metabolic, and MISC = miscellaneous. ^a,b^ Superscripts denote significant differences (*p* < 0.05) within each health category (column).

**Table 9:** Least squares means for total health costs (THC) by herd and parity.

	Parity									
	1		2		3		4		5	
Herd	$\overset{-}{X}$	SE	$\overset{-}{X}$	SE	$\overset{-}{X}$	SE	$\overset{-}{X}$	SE	$\overset{-}{X}$	SE
	-------- $ --------		-------- $ --------		-------- $ --------		-------- $ --------		-------- $ --------	
A	74.60 ^a^	4.11	98.81 ^a^	5.99	118.50 ^ab^	9.18	100.04 ^bc^	16.51	129.18 ^ab^	25.22
B	64.60 ^ab^	4.07	50.07 ^d^	5.62	58.92 ^cde^	8.38	61.82 ^cd^	13.70	37.83 ^c^	19.31
C	53.13 ^c^	3.95	107.61 ^a^	5.43	140.34 ^a^	7.63	176.52 ^a^	12.57	170.09 ^a^	15.86
D	59.57 ^bc^	6.10	75.90 ^bc^	8.69	87.29 ^bc^	13.36	90.00 ^bcd^	30.44	156.17 ^a^	42.45
E	49.76 ^c^	5.41	68.47 ^c^	7.50	72.69 ^cd^	10.26	58.64 ^cd^	17.77	30.67 ^c^	34.66
F	64.81 ^abc^	6.03	92.70 ^ab^	8.40	117.90 ^ab^	12.18	114.53 ^b^	21.52	123.45 ^ab^	31.35
G	29.79 ^d^	5.16	36.11 ^de^	7.45	43.55 ^de^	12.63	59.61 ^bcd^	21.17	23.88 ^c^	36.76
H	23.38 ^d^	6.62	26.86 ^e^	9.17	27.02 ^e^	14.81	21.59 ^d^	28.59	25.60 ^bc^	46.50

^a–e^ Superscripts denote significant differences (*p* < 0.05) between herds within each parity (column).

## Data Availability

The data presented in this study are available on request from the corresponding author.

## References

[1] Rauw W.M., Kanis E., Noordhuizen-Stassen E.N., Grommers F.J. (1998). Undesirable Side Effects of Selection for High Production Efficiency in Farm Animals: A Review. Livest. Prod. Sci..

[2] Haile-Mariam M., Goddard M.E., Haile-Mariam M., Goddard M.E. (2010). Preliminary Genetic Analyses of Voluntarily Supplied Disease Data in Australian Dairy Herds. Anim. Prod. Sci..

[3] Oltenacu P., Broom D. (2010). The Impact of Genetic Selection for Increased Milk Yield on the Welfare of Dairy Cows. Anim. Welf..

[4] Becker J.C., Heins B.J., Hansen L.B. (2012). Costs for Health Care of Holstein Cows Selected for Large versus Small Body Size. J. Dairy Sci..

[5] Mahoney C.B., Hansen L.B., Young C.W., Marx G.D., Reneau J.K. (1986). Health Care of Holsteins Selected for Large or Small Body Size1,2. J. Dairy Sci..

[6] Fessenden B., Weigel D.J., Osterstock J., Galligan D.T., Di Croce F. (2020). Validation of Genomic Predictions for a Lifetime Merit Selection Index for the US Dairy Industry. J. Dairy Sci..

[7] Heins B.J., Hansen L.B., De Vries A. (2012). Survival, Lifetime Production, and Profitability of Normande × Holstein, Montbéliarde × Holstein, and Scandinavian Red × Holstein Crossbreds versus Pure Holsteins. J. Dairy Sci..

[8] Parker Gaddis K.L., Cole J.B., Clay J.S., Maltecca C. (2014). Genomic Selection for Producer-Recorded Health Event Data in US Dairy Cattle. J. Dairy Sci..

[9] Hazel A.R., Heins B.J., Hansen L.B. (2020). Health Treatment Cost, Stillbirth, Survival, and Conformation of Viking Red-, Montbéliarde-, and Holstein-Sired Crossbred Cows Compared with Pure Holstein Cows during Their First 3 Lactations. J. Dairy Sci..

[10] Wenz J.R., Giebel S.K. (2012). Retrospective Evaluation of Health Event Data Recording on 50 Dairies Using Dairy Comp 305. J. Dairy Sci..

[11] USDA (2008). Dairy 2007, Part I: References of Dairy Cattle Health and Management Practices in the United States, 2007.

[12] Heins B.J., Hansen L.B. (2012). Short Communication: Fertility, Somatic Cell Score, and Production of Normande × Holstein, Montbéliarde × Holstein, and Scandinavian Red × Holstein Crossbreds versus Pure Holsteins during Their First 5 Lactations. J. Dairy Sci..

[13] Zwald N.R., Weigel K.A., Chang Y.M., Welper R.D., Clay J.S. (2004). Genetic Selection for Health Traits Using Producer-Recorded Data. I. Incidence Rates, Heritability Estimates, and Sire Breeding Values. J. Dairy Sci..

[14] Hazel A.R., Heins B.J., Hansen L.B. (2017). Production and Calving Traits of Montbéliarde × Holstein and Viking Red × Holstein Cows Compared with Pure Holstein Cows during First Lactation in 8 Commercial Dairy Herds. J. Dairy Sci..

[15] Hazel A.R., Heins B.J., Hansen L.B. (2020). Fertility and 305-Day Production of Viking Red-, Montbéliarde-, and Holstein-Sired Crossbred Cows Compared with Holstein Cows during Their First 3 Lactations in Minnesota Dairy Herds. J. Dairy Sci..

[16] Dechow C.D., Goodling R.C. (2008). Mortality, Culling by Sixty Days in Milk, and Production Profiles in High- and Low-Survival Pennsylvania Herds. J. Dairy Sci..

[17] Parker Gaddis K.L., Cole J.B., Clay J.S., Maltecca C. (2012). Incidence Validation and Relationship Analysis of Producer-Recorded Health Event Data from on-Farm Computer Systems in the United States. J. Dairy Sci..

[18] Vukasinovic N., Bacciu N., Przybyla C.A., Boddhireddy P., DeNise S.K. (2017). Development of Genetic and Genomic Evaluation for Wellness Traits in US Holstein Cows. J. Dairy Sci..

[19] McNeel A.K., Reiter B.C., Weigel D., Osterstock J., Croce F.A.D. (2017). Validation of Genomic Predictions for Wellness Traits in US Holstein Cows. J. Dairy Sci..

[20] Gonzalez-Peña D., Vukasinovic N., Brooker J.J., Przybyla C.A., DeNise S.K. (2019). Genomic Evaluation for Calf Wellness Traits in Holstein Cattle. J. Dairy Sci..

[21] Gonzalez-Peña D., Vukasinovic N., Brooker J.J., Przybyla C.A., Baktula A., DeNise S.K. (2020). Genomic Evaluation for Wellness Traits in US Jersey Cattle. J. Dairy Sci..

[22] Hansen L.B. (2000). Consequences of Selection for Milk Yield from a Geneticist’s Viewpoint. J. Dairy Sci..

[23] Harder B., Bennewitz J., Hinrichs D., Kalm E. (2006). Genetic Parameters for Health Traits and Their Relationship to Different Persistency Traits in German Holstein Dairy Cattle. J. Dairy Sci..

[24] Appuhamy J.A.D.R.N., Cassell B.G., Dechow C.D., Cole J.B. (2007). Phenotypic Relationships of Common Health Disorders in Dairy Cows to Lactation Persistency Estimated from Daily Milk Weights. J. Dairy Sci..

[25] Sundrum A. (2015). Metabolic Disorders in the Transition Period Indicate That the Dairy Cows’ Ability to Adapt Is Overstressed. Animals.

[26] Shim E.H., Shanks R.D., Morin D.E. (2004). Milk Loss and Treatment Costs Associated with Two Treatment Protocols for Clinical Mastitis in Dairy Cows. J. Dairy Sci..

[27] Weigel K.A. (2004). Improving the Reproductive Efficiency of Dairy Cattle through Genetic Selection. J. Dairy Sci..

[28] Antanaitis R., Juozaitienė V., Jonike V., Baumgartner W., Paulauskas A. (2022). Subclinical Mastitis Detected during the Last Gestation Period Can Increase the Risk of Stillbirth in Dairy Calves. Animals.

[29] Sokolov S., Fursova K., Shulcheva I., Nikanova D., Artyemieva O., Kolodina E., Sorokin A., Dzhelyadin T., Shchannikova M., Shepelyakovskaya A. (2021). Comparative Analysis of Milk Microbiomes and Their Association with Bovine Mastitis in Two Farms in Central Russia. Animals.

[30] Sharun K., Dhama K., Tiwari R., Gugjoo M.B., Iqbal Yatoo M., Patel S.K., Pathak M., Karthik K., Khurana S.K., Singh R. (2021). Advances in Therapeutic and Managemental Approaches of Bovine Mastitis: A Comprehensive Review. Vet. Q..

[31] Gussmann M., Steeneveld W., Kirkeby C., Hogeveen H., Farre M., Halasa T. (2019). Economic and Epidemiological Impact of Different Intervention Strategies for Subclinical and Clinical Mastitis. Prev. Vet. Med..

[32] Cobirka M., Tancin V., Slama P. (2020). Epidemiology and Classification of Mastitis. Animals.

[33] Ettema J.F., Santos J.E.P. (2004). Impact of Age at Calving on Lactation, Reproduction, Health, and Income in First-Parity Holsteins on Commercial Farms. J. Dairy Sci..

[34] Guard C.L. The Costs of Common Diseases of Dairy Cattle. https://www.dvm360.com/view/costs-common-diseases-dairy-cattle-proceedings-0.

[35] Jones W.P., Hansen L.B., Chester-Jones H. (1994). Response of Health Care to Selection for Milk Yield of Dairy Cattle1. J. Dairy Sci..

[36] Zwald N.R., Weigel K.A., Chang Y.M., Welper R.D., Clay J.S. (2004). Genetic Selection for Health Traits Using Producer-Recorded Data. II. Genetic Correlations, Disease Probabilities, and Relationships with Existing Traits. J. Dairy Sci..

[37] Hardie L.C., Heins B.J., Dechow C.D. (2021). Genetic Parameters for Stayability of Holsteins in US Organic Herds. J. Dairy Sci..

[38] Hardie L.C., Haagen I.W., Heins B.J., Dechow C.D. (2022). Genetic Parameters and Association of National Evaluations with Breeding Values for Health Traits in US Organic Holstein Cows. J. Dairy Sci..

[39] Koeck A., Miglior F., Kelton D.F., Schenkel F.S. (2012). Health Recording in Canadian Holsteins: Data and Genetic Parameters. J. Dairy Sci..

[40] Leblanc S. (2010). Monitoring Metabolic Health of Dairy Cattle in the Transition Period. J. Reprod. Dev..

[41] Green M.J., Bradley A.J., Medley G.F., Browne W.J. (2007). Cow, Farm, and Management Factors During the Dry Period That Determine the Rate of Clinical Mastitis After Calving. J. Dairy Sci..

[42] de Haas Y., Barkema H.W., Veerkamp R.F. (2002). The Effect of Pathogen-Specific Clinical Mastitis on the Lactation Curve for Somatic Cell Count. J. Dairy Sci..

[43] Lago A., Godden S.M., Bey R., Ruegg P.L., Leslie K. (2011). The Selective Treatment of Clinical Mastitis Based on On-Farm Culture Results: I. Effects on Antibiotic Use, Milk Withholding Time, and Short-Term Clinical and Bacteriological Outcomes. J. Dairy Sci..

[44] Hutchinson J.L. Trouble-Shooting Infertility Problems in Cattle. https://extension.psu.edu/trouble-shooting-infertility-problems-in-cattle.

[45] Esposito G., Irons P.C., Webb E.C., Chapwanya A. (2014). Interactions between Negative Energy Balance, Metabolic Diseases, Uterine Health and Immune Response in Transition Dairy Cows. Anim. Reprod. Sci..

[46] Heins B.J., Pereira G.M., Sharpe K.T. Precision Technologies to Improve Dairy Grazing Systems. JDS Commun..

[47] Pereira G.M., Heins B.J. (2019). Activity and Rumination of Holstein and Crossbred Cows in an Organic Grazing and Low-Input Conventional Dairy Herd. Transl. Anim. Sci..

[48] Pereira G.M., Heins B.J., Endres M.I. (2020). Estrous Detection with an Activity and Rumination Monitoring System in an Organic Grazing and a Low-Input Conventional Dairy Herd. Anim. Reprod. Sci..

[49] Minegishi K., Heins B.J., Pereira G.M. (2019). Peri-Estrus Activity and Rumination Time and Its Application to Estrus Prediction: Evidence from Dairy Herds under Organic Grazing and Low-Input Conventional Production. Livest. Sci..

